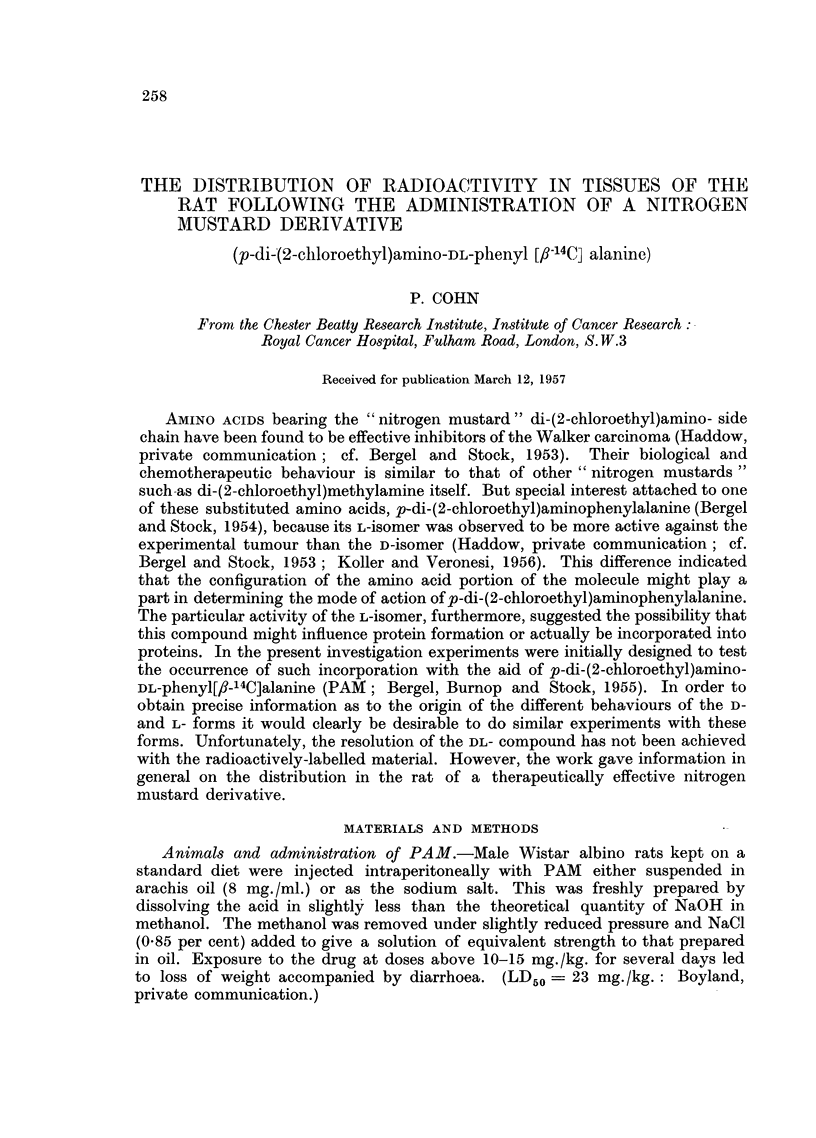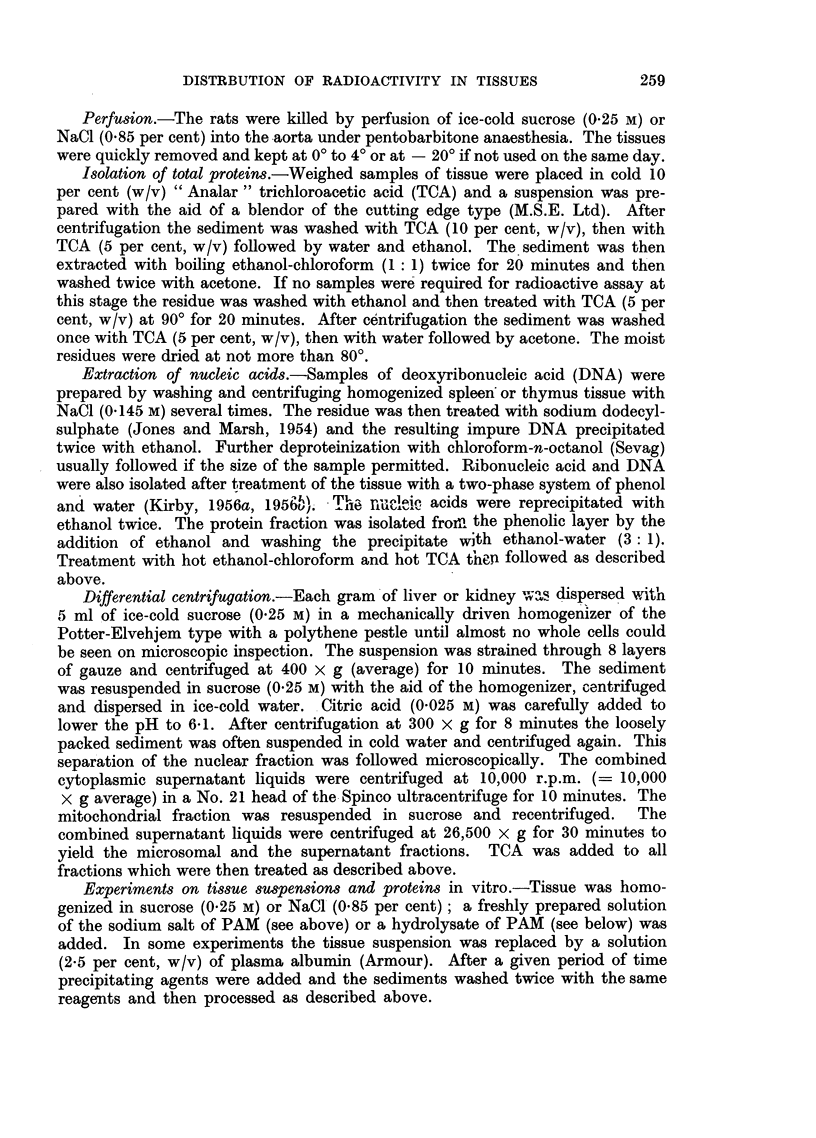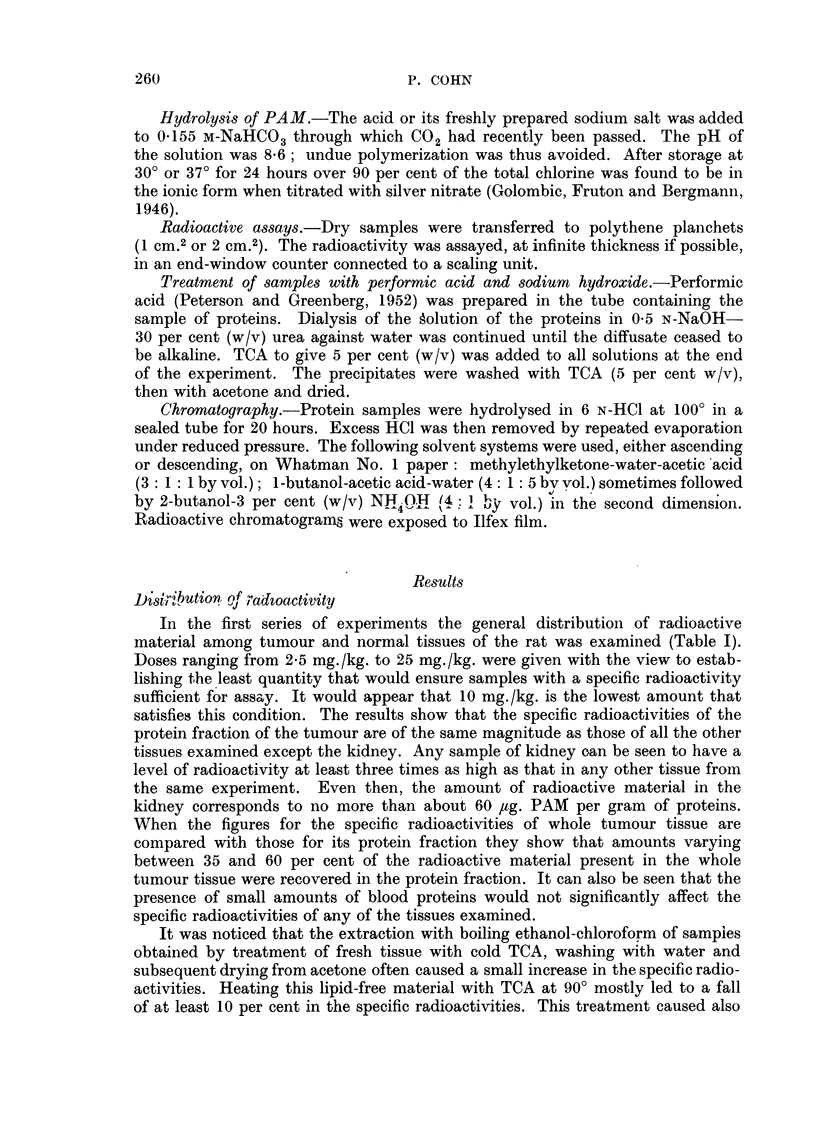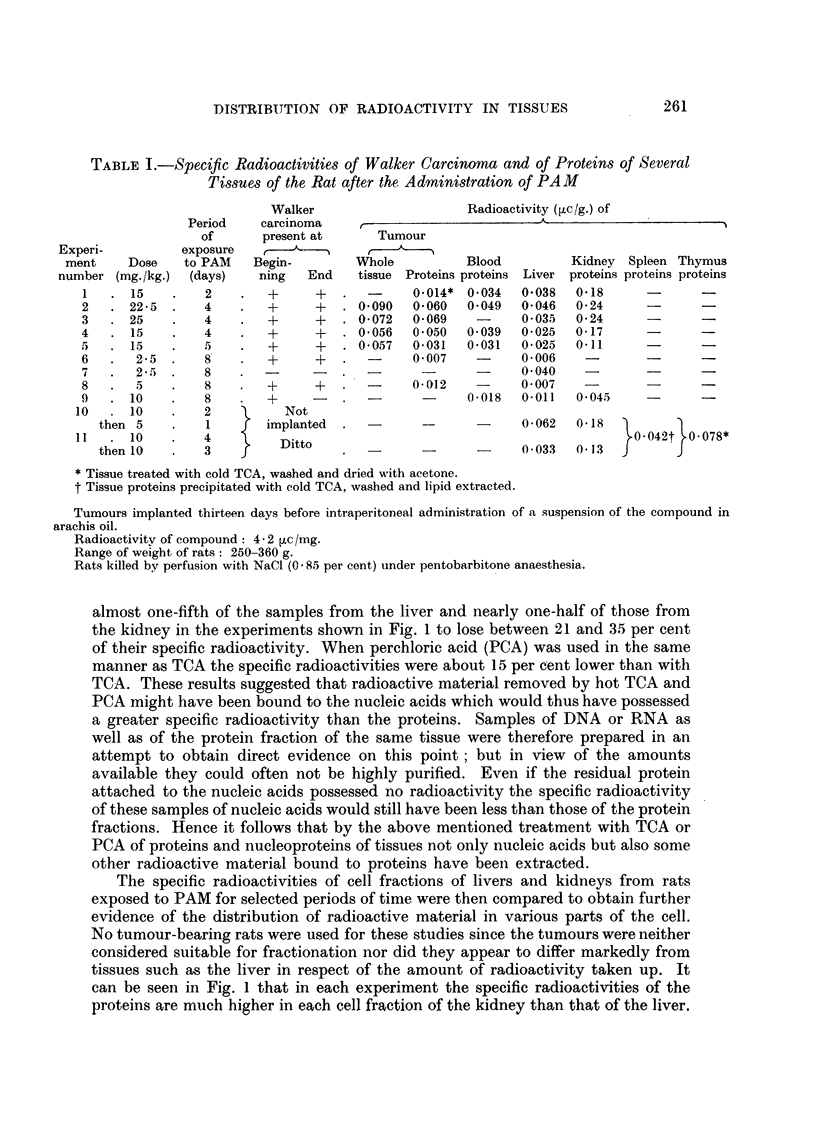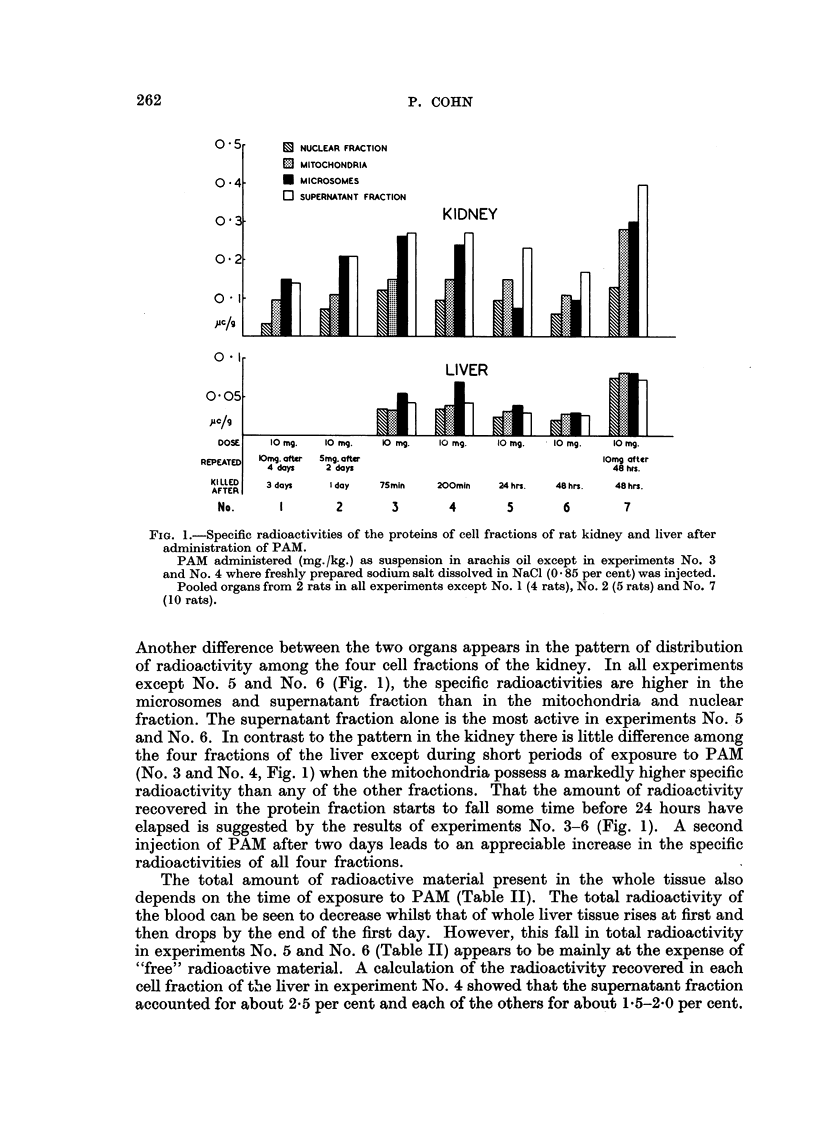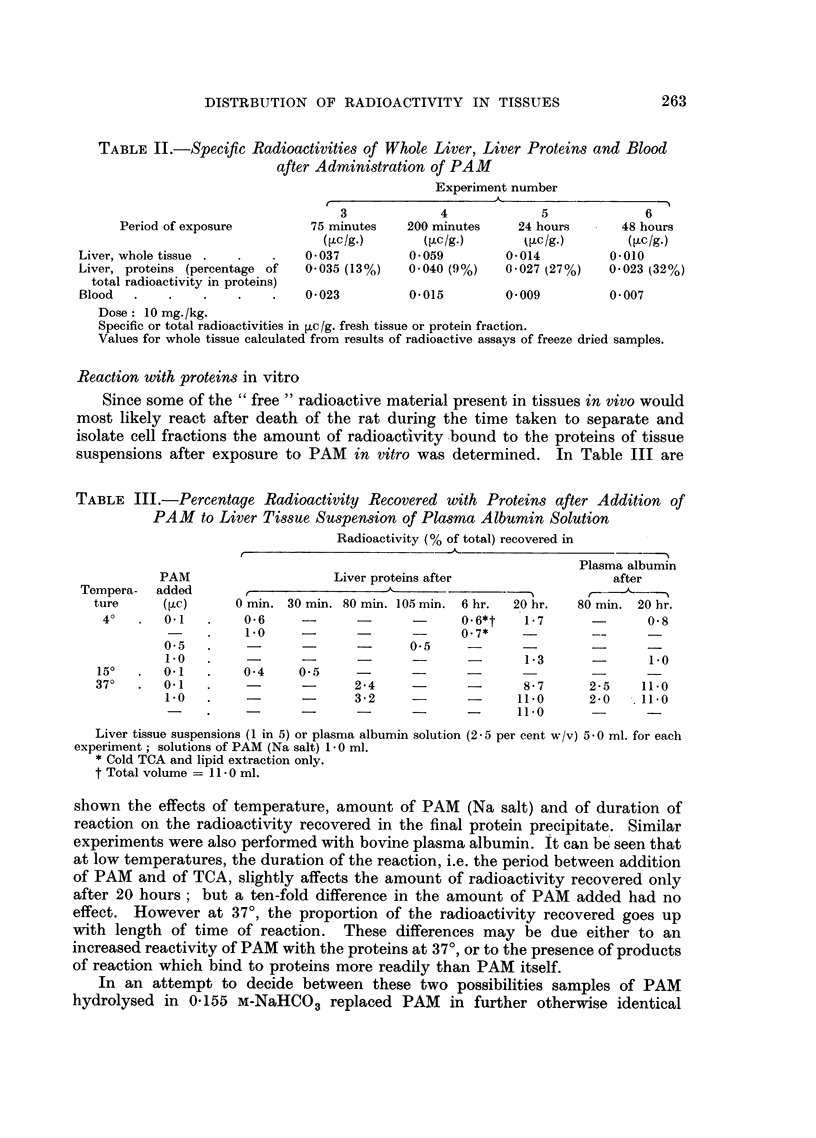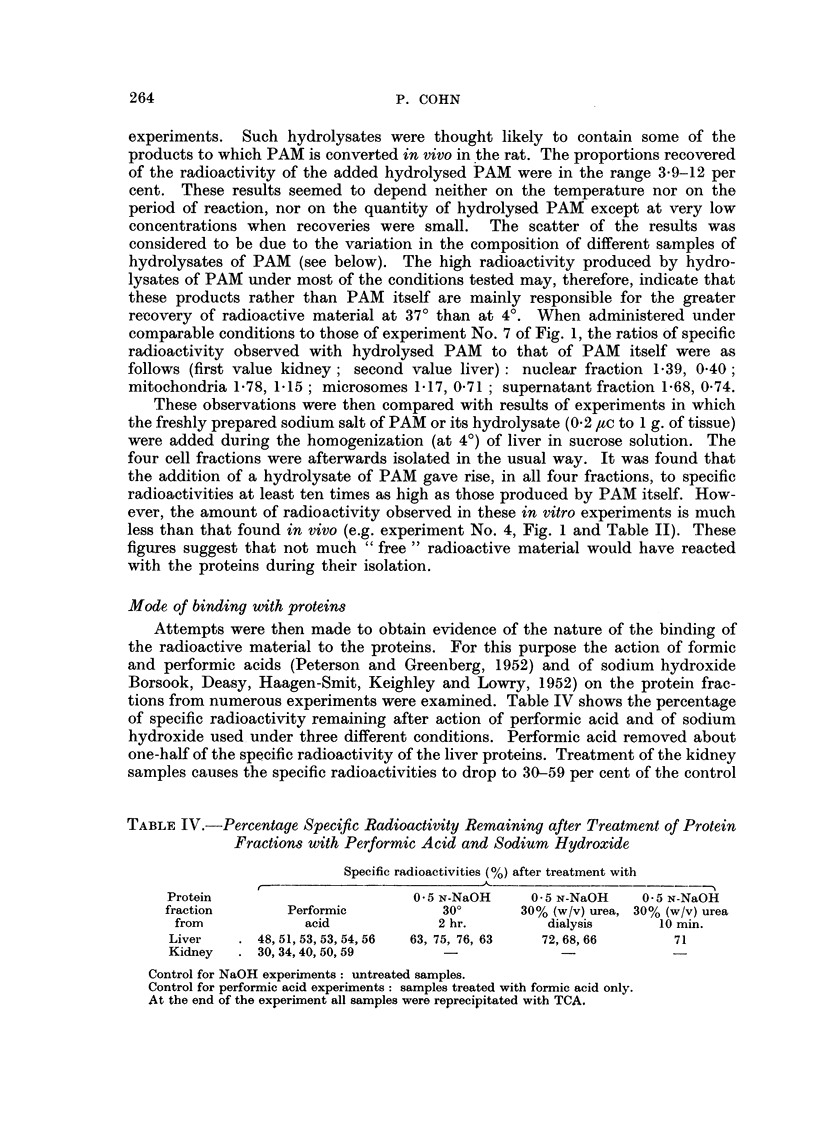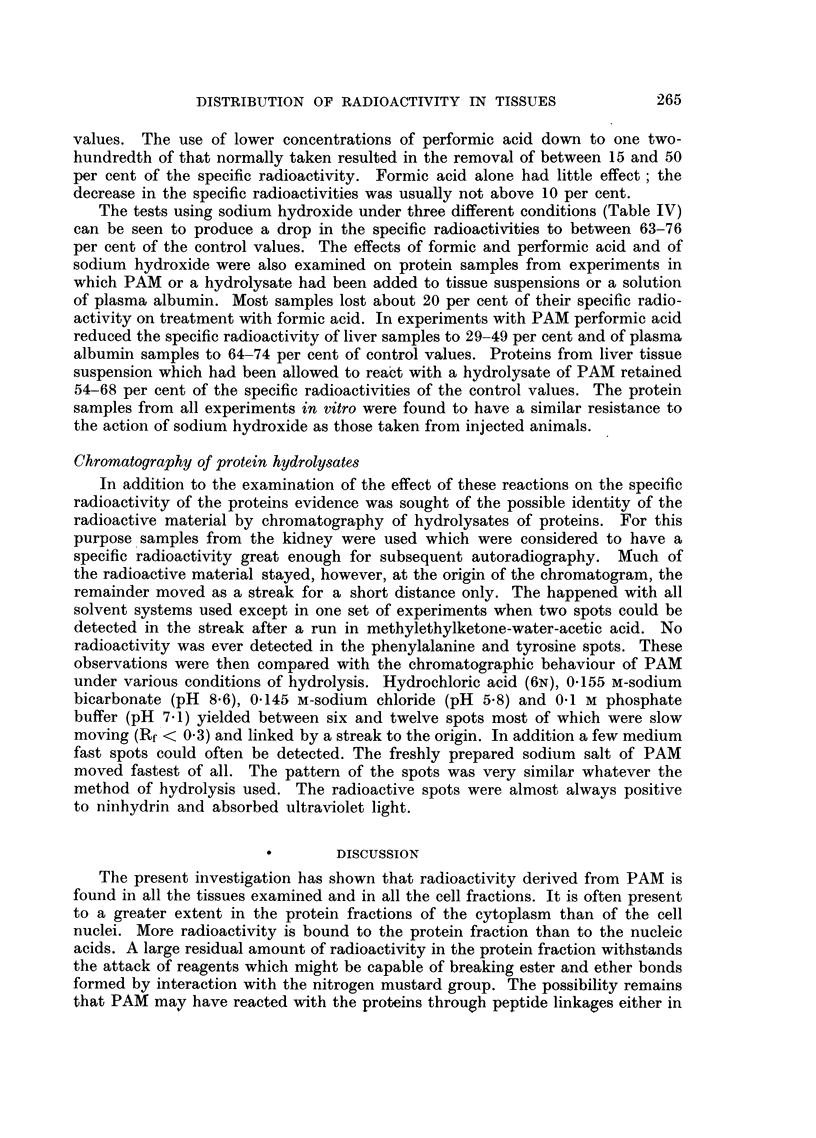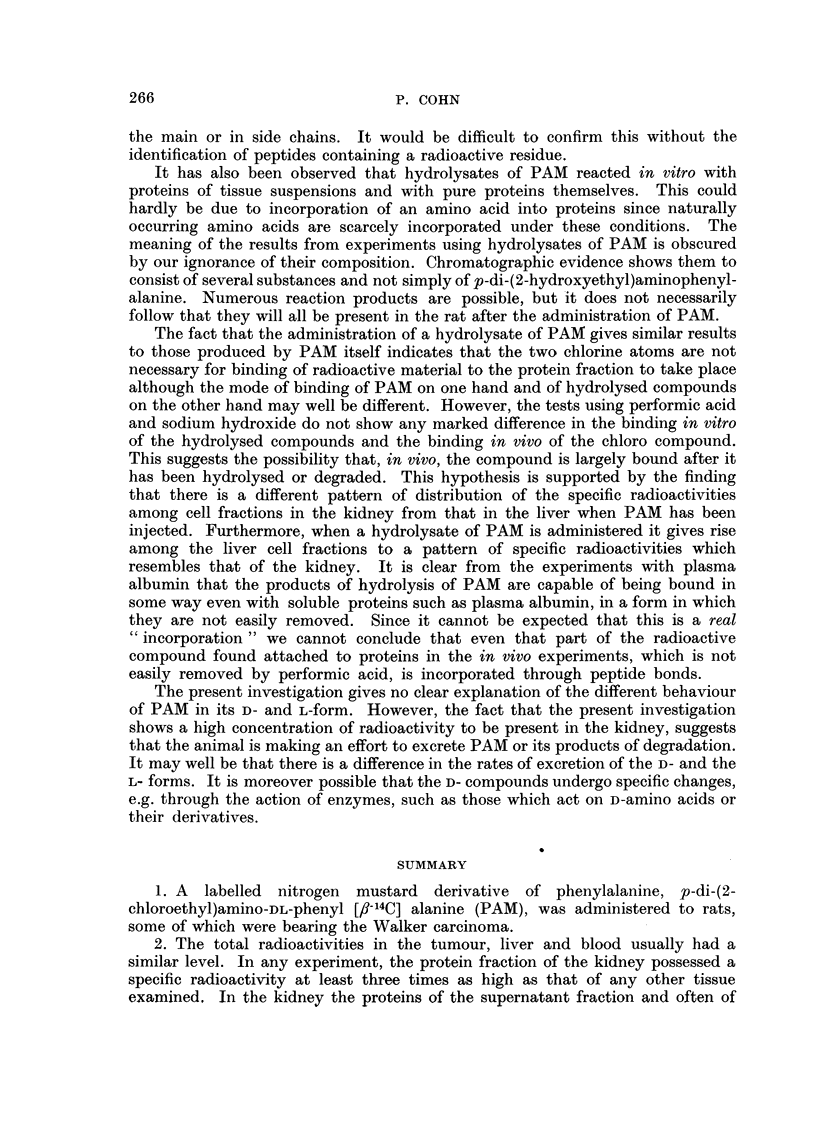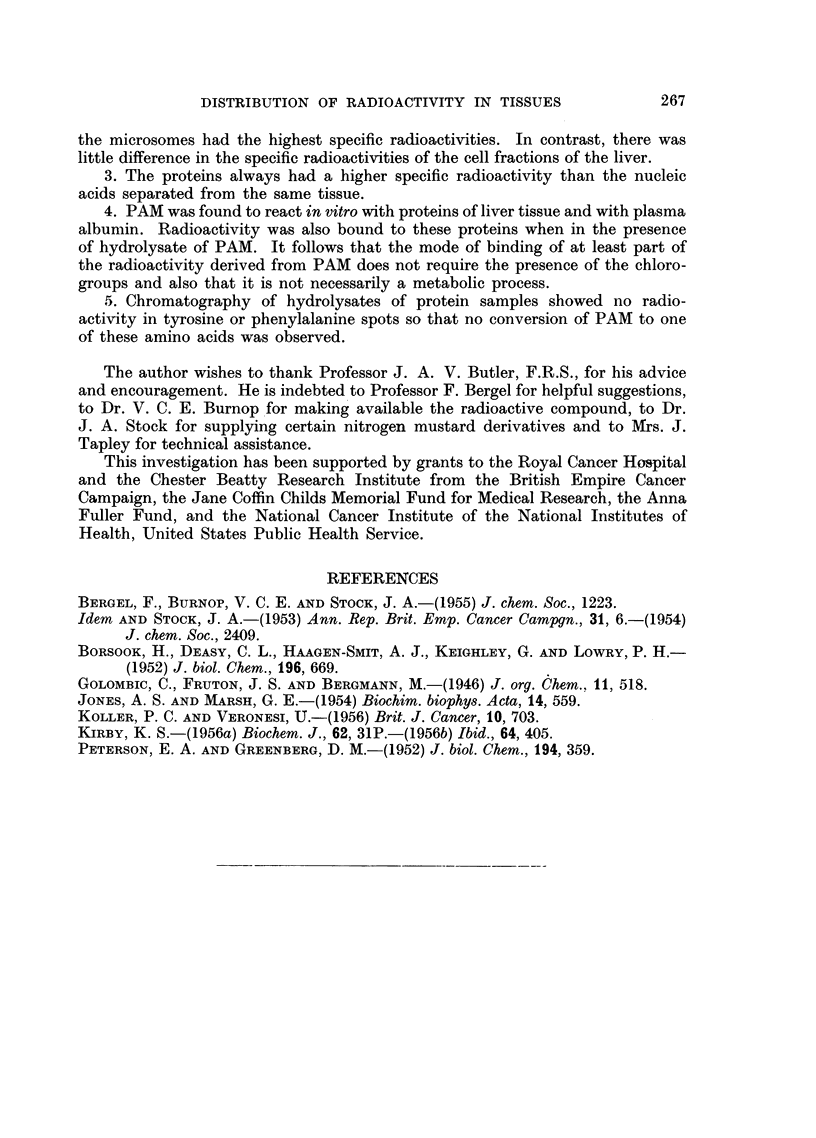# The Distribution of Radioactivity in Tissues of the Rat Following the Administration of a Nitrogen Mustard Derivative

**DOI:** 10.1038/bjc.1957.33

**Published:** 1957-06

**Authors:** P. Cohn


### THE DISTRIBUTION OF RADIOACTIVITY IN TISSUES OF THE RAT FOLLOWING THE ADMINISTRATION OF A NITROGEN MUSTARD DERIVATIVE

(*p*-di-(2-chloroethyl)amino-DL-phenyl [$\beta$-$^{14}$C] alanine)

P. COHN

*From the Chester Beatty Research Institute, Institute of Cancer Research : Royal Cancer Hospital, Fulham Road, London, S.W.3*

Received for publication March 12, 1957

AMINO ACIDS bearing the "nitrogen mustard" di-(2-chloroethyl)amino- side chain have been found to be effective inhibitors of the Walker carcinoma (Haddow, private communication ; cf. Bergel and Stock, 1953). Their biological and chemotherapeutic behaviour is similar to that of other "nitrogen mustards" such as di-(2-chloroethyl)methylamine itself. But special interest attached to one of these substituted amino acids, *p*-di-(2-chloroethyl)aminophenylalanine (Bergel and Stock, 1954), because its L-isomer was observed to be more active against the experimental tumour than the D-isomer (Haddow, private communication ; cf. Bergel and Stock, 1953 ; Koller and Veronesi, 1956). This difference indicated that the configuration of the amino acid portion of the molecule might play a part in determining the mode of action of *p*-di-(2-chloroethyl)aminophenylalanine. The particular activity of the L-isomer, furthermore, suggested the possibility that this compound might influence protein formation or actually be incorporated into proteins. In the present investigation experiments were initially designed to test the occurrence of such incorporation with the aid of *p*-di-(2-chloroethyl)amino-DL-phenyl[$\beta$-$^{14}$C]alanine (PAM ; Bergel, Burnop and Stock, 1955). In order to obtain precise information as to the origin of the different behaviours of the D- and L- forms it would clearly be desirable to do similar experiments with these forms. Unfortunately, the resolution of the DL- compound has not been achieved with the radioactively-labelled material. However, the work gave information in general on the distribution in the rat of a therapeutically effective nitrogen mustard derivative.

### MATERIALS AND METHODS

*Animals and administration of PAM.*—Male Wistar albino rats kept on a standard diet were injected intraperitoneally with PAM either suspended in arachis oil (8 mg./ml.) or as the sodium salt. This was freshly prepared by dissolving the acid in slightly less than the theoretical quantity of NaOH in methanol. The methanol was removed under slightly reduced pressure and NaCl (0·85 per cent) added to give a solution of equivalent strength to that prepared in oil. Exposure to the drug at doses above 10–15 mg./kg. for several days led to loss of weight accompanied by diarrhoea. ($LD_{50}$ = 23 mg./kg. : Boyland, private communication.)

*Perfusion.*—The rats were killed by perfusion of ice-cold sucrose (0·25 M) or NaCl (0·85 per cent) into the aorta under pentobarbitone anaesthesia. The tissues were quickly removed and kept at 0° to 4° or at — 20° if not used on the same day.

*Isolation of total proteins.*—Weighed samples of tissue were placed in cold 10 per cent (w/v) " Analar " trichloroacetic acid (TCA) and a suspension was prepared with the aid of a blendor of the cutting edge type (M.S.E. Ltd). After centrifugation the sediment was washed with TCA (10 per cent, w/v), then with TCA (5 per cent, w/v) followed by water and ethanol. The sediment was then extracted with boiling ethanol-chloroform (1 : 1) twice for 20 minutes and then washed twice with acetone. If no samples were required for radioactive assay at this stage the residue was washed with ethanol and then treated with TCA (5 per cent, w/v) at 90° for 20 minutes. After centrifugation the sediment was washed once with TCA (5 per cent, w/v), then with water followed by acetone. The moist residues were dried at not more than 80°.

*Extraction of nucleic acids.*—Samples of deoxyribonucleic acid (DNA) were prepared by washing and centrifuging homogenized spleen or thymus tissue with NaCl (0·145 M) several times. The residue was then treated with sodium dodecylsulphate (Jones and Marsh, 1954) and the resulting impure DNA precipitated twice with ethanol. Further deproteinization with chloroform-*n*-octanol (Sevag) usually followed if the size of the sample permitted. Ribonucleic acid and DNA were also isolated after treatment of the tissue with a two-phase system of phenol and water (Kirby, 1956*a*, 1956*b*). The nucleic acids were reprecipitated with ethanol twice. The protein fraction was isolated from the phenolic layer by the addition of ethanol and washing the precipitate with ethanol-water (3 : 1). Treatment with hot ethanol-chloroform and hot TCA then followed as described above.

*Differential centrifugation.*—Each gram of liver or kidney was dispersed with 5 ml of ice-cold sucrose (0·25 M) in a mechanically driven homogenizer of the Potter-Elvehjem type with a polythene pestle until almost no whole cells could be seen on microscopic inspection. The suspension was strained through 8 layers of gauze and centrifuged at 400 × g (average) for 10 minutes. The sediment was resuspended in sucrose (0·25 M) with the aid of the homogenizer, centrifuged and dispersed in ice-cold water. Citric acid (0·025 M) was carefully added to lower the pH to 6·1. After centrifugation at 300 × g for 8 minutes the loosely packed sediment was often suspended in cold water and centrifuged again. This separation of the nuclear fraction was followed microscopically. The combined cytoplasmic supernatant liquids were centrifuged at 10,000 r.p.m. (= 10,000 × g average) in a No. 21 head of the Spinco ultracentrifuge for 10 minutes. The mitochondrial fraction was resuspended in sucrose and recentrifuged. The combined supernatant liquids were centrifuged at 26,500 × g for 30 minutes to yield the microsomal and the supernatant fractions. TCA was added to all fractions which were then treated as described above.

*Experiments on tissue suspensions and proteins* in vitro.—Tissue was homogenized in sucrose (0·25 M) or NaCl (0·85 per cent) ; a freshly prepared solution of the sodium salt of PAM (see above) or a hydrolysate of PAM (see below) was added. In some experiments the tissue suspension was replaced by a solution (2·5 per cent, w/v) of plasma albumin (Armour). After a given period of time precipitating agents were added and the sediments washed twice with the same reagents and then processed as described above.

*Hydrolysis of PAM.*—The acid or its freshly prepared sodium salt was added to 0·155 M-$NaHCO_3$ through which $CO_2$ had recently been passed. The pH of the solution was 8·6 ; undue polymerization was thus avoided. After storage at 30° or 37° for 24 hours over 90 per cent of the total chlorine was found to be in the ionic form when titrated with silver nitrate (Golombic, Fruton and Bergmann, 1946).

*Radioactive assays.*—Dry samples were transferred to polythene planchets (1 cm.$^2$ or 2 cm.$^2$). The radioactivity was assayed, at infinite thickness if possible, in an end-window counter connected to a scaling unit.

*Treatment of samples with performic acid and sodium hydroxide.*—Performic acid (Peterson and Greenberg, 1952) was prepared in the tube containing the sample of proteins. Dialysis of the solution of the proteins in 0·5 N-NaOH—30 per cent (w/v) urea against water was continued until the diffusate ceased to be alkaline. TCA to give 5 per cent (w/v) was added to all solutions at the end of the experiment. The precipitates were washed with TCA (5 per cent w/v), then with acetone and dried.

*Chromatography.*—Protein samples were hydrolysed in 6 N-HCl at 100° in a sealed tube for 20 hours. Excess HCl was then removed by repeated evaporation under reduced pressure. The following solvent systems were used, either ascending or descending, on Whatman No. 1 paper : methylethylketone-water-acetic acid (3 : 1 : 1 by vol.) ; 1-butanol-acetic acid-water (4 : 1 : 5 by vol.) sometimes followed by 2-butanol-3 per cent (w/v) $NH_4OH$ (4 : 1 by vol.) in the second dimension. Radioactive chromatograms were exposed to Ilfex film.

### *Results*

### *Distribution of radioactivity*

In the first series of experiments the general distribution of radioactive material among tumour and normal tissues of the rat was examined (Table I). Doses ranging from 2·5 mg./kg. to 25 mg./kg. were given with the view to establishing the least quantity that would ensure samples with a specific radioactivity sufficient for assay. It would appear that 10 mg./kg. is the lowest amount that satisfies this condition. The results show that the specific radioactivities of the protein fraction of the tumour are of the same magnitude as those of all the other tissues examined except the kidney. Any sample of kidney can be seen to have a level of radioactivity at least three times as high as that in any other tissue from the same experiment. Even then, the amount of radioactive material in the kidney corresponds to no more than about 60 $\mu$g. PAM per gram of proteins. When the figures for the specific radioactivities of whole tumour tissue are compared with those for its protein fraction they show that amounts varying between 35 and 60 per cent of the radioactive material present in the whole tumour tissue were recovered in the protein fraction. It can also be seen that the presence of small amounts of blood proteins would not significantly affect the specific radioactivities of any of the tissues examined.

It was noticed that the extraction with boiling ethanol-chloroform of samples obtained by treatment of fresh tissue with cold TCA, washing with water and subsequent drying from acetone often caused a small increase in the specific radioactivities. Heating this lipid-free material with TCA at 90° mostly led to a fall of at least 10 per cent in the specific radioactivities. This treatment caused also

TABLE I.—*Specific Radioactivities of Walker Carcinoma and of Proteins of Several Tissues of the Rat after the Administration of PAM*

| Experiment number | Dose (mg./kg.) | Period of exposure to PAM (days) | Walker carcinoma present at: Beginning | Walker carcinoma present at: End | Radioactivity (μc/g.) of: Tumour: Whole tissue | Tumour: Proteins | Blood proteins | Liver | Kidney proteins | Spleen proteins | Thymus proteins |
|---|---|---|---|---|---|---|---|---|---|---|---|
| 1 | 15 | 2 | + | + | — | 0·014* | 0·034 | 0·038 | 0·18 | — | — |
| 2 | 22·5 | 4 | + | + | 0·090 | 0·060 | 0·049 | 0·046 | 0·24 | — | — |
| 3 | 25 | 4 | + | + | 0·072 | 0·069 | — | 0·035 | 0·24 | — | — |
| 4 | 15 | 4 | + | + | 0·056 | 0·050 | 0·039 | 0·025 | 0·17 | — | — |
| 5 | 15 | 5 | + | + | 0·057 | 0·031 | 0·031 | 0·025 | 0·11 | — | — |
| 6 | 2·5 | 8 | + | + | — | 0·007 | — | 0·006 | — | — | — |
| 7 | 2·5 | 8 | — | — | — | — | — | 0·040 | — | — | — |
| 8 | 5 | 8 | + | + | — | 0·012 | — | 0·007 | — | — | — |
| 9 | 10 | 8 | + | — | — | — | 0·018 | 0·011 | 0·045 | — | — |
| 10 | 10 then 5 | 2 then 1 | Not implanted | | — | — | — | 0·062 | 0·18 | 0·042† (10 and 11) | 0·078* (10 and 11) |
| 11 | 10 then 10 | 4 then 3 | Ditto | | — | — | — | 0·033 | 0·13 | | |

* Tissue treated with cold TCA, washed and dried with acetone.
† Tissue proteins precipitated with cold TCA, washed and lipid extracted.

Tumours implanted thirteen days before intraperitoneal administration of a suspension of the compound in arachis oil.
Radioactivity of compound : 4·2 μc/mg.
Range of weight of rats : 250–360 g.
Rats killed by perfusion with NaCl (0·85 per cent) under pentobarbitone anaesthesia.

almost one-fifth of the samples from the liver and nearly one-half of those from the kidney in the experiments shown in Fig. 1 to lose between 21 and 35 per cent of their specific radioactivity. When perchloric acid (PCA) was used in the same manner as TCA the specific radioactivities were about 15 per cent lower than with TCA. These results suggested that radioactive material removed by hot TCA and PCA might have been bound to the nucleic acids which would thus have possessed a greater specific radioactivity than the proteins. Samples of DNA or RNA as well as of the protein fraction of the same tissue were therefore prepared in an attempt to obtain direct evidence on this point ; but in view of the amounts available they could often not be highly purified. Even if the residual protein attached to the nucleic acids possessed no radioactivity the specific radioactivity of these samples of nucleic acids would still have been less than those of the protein fractions. Hence it follows that by the above mentioned treatment with TCA or PCA of proteins and nucleoproteins of tissues not only nucleic acids but also some other radioactive material bound to proteins have been extracted.

The specific radioactivities of cell fractions of livers and kidneys from rats exposed to PAM for selected periods of time were then compared to obtain further evidence of the distribution of radioactive material in various parts of the cell. No tumour-bearing rats were used for these studies since the tumours were neither considered suitable for fractionation nor did they appear to differ markedly from tissues such as the liver in respect of the amount of radioactivity taken up. It can be seen in Fig. 1 that in each experiment the specific radioactivities of the proteins are much higher in each cell fraction of the kidney than that of the liver.

Fig. 1.—Specific radioactivities of the proteins of cell fractions of rat kidney and liver after administration of PAM.

PAM administered (mg./kg.) as suspension in arachis oil except in experiments No. 3 and No. 4 where freshly prepared sodium salt dissolved in NaCl (0·85 per cent) was injected.

Pooled organs from 2 rats in all experiments except No. 1 (4 rats), No. 2 (5 rats) and No. 7 (10 rats).

Another difference between the two organs appears in the pattern of distribution of radioactivity among the four cell fractions of the kidney. In all experiments except No. 5 and No. 6 (Fig. 1), the specific radioactivities are higher in the microsomes and supernatant fraction than in the mitochondria and nuclear fraction. The supernatant fraction alone is the most active in experiments No. 5 and No. 6. In contrast to the pattern in the kidney there is little difference among the four fractions of the liver except during short periods of exposure to PAM (No. 3 and No. 4, Fig. 1) when the mitochondria possess a markedly higher specific radioactivity than any of the other fractions. That the amount of radioactivity recovered in the protein fraction starts to fall some time before 24 hours have elapsed is suggested by the results of experiments No. 3–6 (Fig. 1). A second injection of PAM after two days leads to an appreciable increase in the specific radioactivities of all four fractions.

The total amount of radioactive material present in the whole tissue also depends on the time of exposure to PAM (Table II). The total radioactivity of the blood can be seen to decrease whilst that of whole liver tissue rises at first and then drops by the end of the first day. However, this fall in total radioactivity in experiments No. 5 and No. 6 (Table II) appears to be mainly at the expense of "free" radioactive material. A calculation of the radioactivity recovered in each cell fraction of the liver in experiment No. 4 showed that the supernatant fraction accounted for about 2·5 per cent and each of the others for about 1·5–2·0 per cent.

TABLE II.—*Specific Radioactivities of Whole Liver, Liver Proteins and Blood after Administration of PAM*

| | Experiment number | | | |
|---|---|---|---|---|
| Period of exposure | 3<br>75 minutes<br>(μc/g.) | 4<br>200 minutes<br>(μc/g.) | 5<br>24 hours<br>(μc/g.) | 6<br>48 hours<br>(μc/g.) |
| Liver, whole tissue . . . | 0·037 | 0·059 | 0·014 | 0·010 |
| Liver, proteins (percentage of total radioactivity in proteins) | 0·035 (13%) | 0·040 (9%) | 0·027 (27%) | 0·023 (32%) |
| Blood . . . . . | 0·023 | 0·015 | 0·009 | 0·007 |

Dose : 10 mg./kg.
Specific or total radioactivities in μc/g. fresh tissue or protein fraction.
Values for whole tissue calculated from results of radioactive assays of freeze dried samples.

*Reaction with proteins* in vitro

Since some of the " free " radioactive material present in tissues *in vivo* would most likely react after death of the rat during the time taken to separate and isolate cell fractions the amount of radioactivity bound to the proteins of tissue suspensions after exposure to PAM *in vitro* was determined. In Table III are

TABLE III.—*Percentage Radioactivity Recovered with Proteins after Addition of PAM to Liver Tissue Suspension of Plasma Albumin Solution*

| | | Radioactivity (% of total) recovered in | | | | | | | |
|---|---|---|---|---|---|---|---|---|---|
| | | Liver proteins after | | | | | | Plasma albumin after | |
| Temperature | PAM added (μc) | 0 min. | 30 min. | 80 min. | 105 min. | 6 hr. | 20 hr. | 80 min. | 20 hr. |
| 4° . | 0·1 . | 0·6 | — | — | — | 0·6*† | 1·7 | — | 0·8 |
| | — . | 1·0 | — | — | — | 0·7* | — | — | — |
| | 0·5 . | — | — | — | 0·5 | — | — | — | — |
| | 1·0 . | — | — | — | — | — | 1·3 | — | 1·0 |
| 15° . | 0·1 . | 0·4 | 0·5 | — | — | — | — | — | — |
| 37° . | 0·1 . | — | — | 2·4 | — | — | 8·7 | 2·5 | 11·0 |
| | 1·0 . | — | — | 3·2 | — | — | 11·0 | 2·0 | 11·0 |
| | — . | — | — | — | — | — | 11·0 | — | — |

Liver tissue suspensions (1 in 5) or plasma albumin solution (2·5 per cent w/v) 5·0 ml. for each experiment ; solutions of PAM (Na salt) 1·0 ml.
* Cold TCA and lipid extraction only.
† Total volume = 11·0 ml.

shown the effects of temperature, amount of PAM (Na salt) and of duration of reaction on the radioactivity recovered in the final protein precipitate. Similar experiments were also performed with bovine plasma albumin. It can be seen that at low temperatures, the duration of the reaction, i.e. the period between addition of PAM and of TCA, slightly affects the amount of radioactivity recovered only after 20 hours ; but a ten-fold difference in the amount of PAM added had no effect. However at 37°, the proportion of the radioactivity recovered goes up with length of time of reaction. These differences may be due either to an increased reactivity of PAM with the proteins at 37°, or to the presence of products of reaction which bind to proteins more readily than PAM itself.

In an attempt to decide between these two possibilities samples of PAM hydrolysed in 0·155 M-$NaHCO_3$ replaced PAM in further otherwise identical experiments. Such hydrolysates were thought likely to contain some of the products to which PAM is converted *in vivo* in the rat. The proportions recovered of the radioactivity of the added hydrolysed PAM were in the range 3·9–12 per cent. These results seemed to depend neither on the temperature nor on the period of reaction, nor on the quantity of hydrolysed PAM except at very low concentrations when recoveries were small. The scatter of the results was considered to be due to the variation in the composition of different samples of hydrolysates of PAM (see below). The high radioactivity produced by hydrolysates of PAM under most of the conditions tested may, therefore, indicate that these products rather than PAM itself are mainly responsible for the greater recovery of radioactive material at 37° than at 4°. When administered under comparable conditions to those of experiment No. 7 of Fig. 1, the ratios of specific radioactivity observed with hydrolysed PAM to that of PAM itself were as follows (first value kidney ; second value liver) : nuclear fraction 1·39, 0·40 ; mitochondria 1·78, 1·15 ; microsomes 1·17, 0·71 ; supernatant fraction 1·68, 0·74.

These observations were then compared with results of experiments in which the freshly prepared sodium salt of PAM or its hydrolysate (0·2 $\mu$c to 1 g. of tissue) were added during the homogenization (at 4°) of liver in sucrose solution. The four cell fractions were afterwards isolated in the usual way. It was found that the addition of a hydrolysate of PAM gave rise, in all four fractions, to specific radioactivities at least ten times as high as those produced by PAM itself. However, the amount of radioactivity observed in these *in vitro* experiments is much less than that found *in vivo* (e.g. experiment No. 4, Fig. 1 and Table II). These figures suggest that not much " free " radioactive material would have reacted with the proteins during their isolation.

*Mode of binding with proteins*

Attempts were then made to obtain evidence of the nature of the binding of the radioactive material to the proteins. For this purpose the action of formic and performic acids (Peterson and Greenberg, 1952) and of sodium hydroxide Borsook, Deasy, Haagen-Smit, Keighley and Lowry, 1952) on the protein fractions from numerous experiments were examined. Table IV shows the percentage of specific radioactivity remaining after action of performic acid and of sodium hydroxide used under three different conditions. Performic acid removed about one-half of the specific radioactivity of the liver proteins. Treatment of the kidney samples causes the specific radioactivities to drop to 30–59 per cent of the control

TABLE IV.—*Percentage Specific Radioactivity Remaining after Treatment of Protein Fractions with Performic Acid and Sodium Hydroxide*

| Protein fraction from | Specific radioactivities (%) after treatment with | | | |
|---|---|---|---|---|
| | Performic acid | 0·5 N-NaOH 30° 2 hr. | 0·5 N-NaOH 30% (w/v) urea, dialysis | 0·5 N-NaOH 30% (w/v) urea 10 min. |
| Liver . | 48, 51, 53, 53, 54, 56 | 63, 75, 76, 63 | 72, 68, 66 | 71 |
| Kidney . | 30, 34, 40, 50, 59 | — | — | — |

Control for NaOH experiments : untreated samples.
Control for performic acid experiments : samples treated with formic acid only.
At the end of the experiment all samples were reprecipitated with TCA.

values. The use of lower concentrations of performic acid down to one two-hundredth of that normally taken resulted in the removal of between 15 and 50 per cent of the specific radioactivity. Formic acid alone had little effect ; the decrease in the specific radioactivities was usually not above 10 per cent.

The tests using sodium hydroxide under three different conditions (Table IV) can be seen to produce a drop in the specific radioactivities to between 63–76 per cent of the control values. The effects of formic and performic acid and of sodium hydroxide were also examined on protein samples from experiments in which PAM or a hydrolysate had been added to tissue suspensions or a solution of plasma albumin. Most samples lost about 20 per cent of their specific radioactivity on treatment with formic acid. In experiments with PAM performic acid reduced the specific radioactivity of liver samples to 29–49 per cent and of plasma albumin samples to 64–74 per cent of control values. Proteins from liver tissue suspension which had been allowed to react with a hydrolysate of PAM retained 54–68 per cent of the specific radioactivities of the control values. The protein samples from all experiments *in vitro* were found to have a similar resistance to the action of sodium hydroxide as those taken from injected animals.

*Chromatography of protein hydrolysates*

In addition to the examination of the effect of these reactions on the specific radioactivity of the proteins evidence was sought of the possible identity of the radioactive material by chromatography of hydrolysates of proteins. For this purpose samples from the kidney were used which were considered to have a specific radioactivity great enough for subsequent autoradiography. Much of the radioactive material stayed, however, at the origin of the chromatogram, the remainder moved as a streak for a short distance only. The happened with all solvent systems used except in one set of experiments when two spots could be detected in the streak after a run in methylethylketone-water-acetic acid. No radioactivity was ever detected in the phenylalanine and tyrosine spots. These observations were then compared with the chromatographic behaviour of PAM under various conditions of hydrolysis. Hydrochloric acid (6N), 0·155 M-sodium bicarbonate (pH 8·6), 0·145 M-sodium chloride (pH 5·8) and 0·1 M phosphate buffer (pH 7·1) yielded between six and twelve spots most of which were slow moving ($R_f < 0{\cdot}3$) and linked by a streak to the origin. In addition a few medium fast spots could often be detected. The freshly prepared sodium salt of PAM moved fastest of all. The pattern of the spots was very similar whatever the method of hydrolysis used. The radioactive spots were almost always positive to ninhydrin and absorbed ultraviolet light.

### DISCUSSION

The present investigation has shown that radioactivity derived from PAM is found in all the tissues examined and in all the cell fractions. It is often present to a greater extent in the protein fractions of the cytoplasm than of the cell nuclei. More radioactivity is bound to the protein fraction than to the nucleic acids. A large residual amount of radioactivity in the protein fraction withstands the attack of reagents which might be capable of breaking ester and ether bonds formed by interaction with the nitrogen mustard group. The possibility remains that PAM may have reacted with the proteins through peptide linkages either in the main or in side chains. It would be difficult to confirm this without the identification of peptides containing a radioactive residue.

It has also been observed that hydrolysates of PAM reacted *in vitro* with proteins of tissue suspensions and with pure proteins themselves. This could hardly be due to incorporation of an amino acid into proteins since naturally occurring amino acids are scarcely incorporated under these conditions. The meaning of the results from experiments using hydrolysates of PAM is obscured by our ignorance of their composition. Chromatographic evidence shows them to consist of several substances and not simply of *p*-di-(2-hydroxyethyl)aminophenylalanine. Numerous reaction products are possible, but it does not necessarily follow that they will all be present in the rat after the administration of PAM.

The fact that the administration of a hydrolysate of PAM gives similar results to those produced by PAM itself indicates that the two chlorine atoms are not necessary for binding of radioactive material to the protein fraction to take place although the mode of binding of PAM on one hand and of hydrolysed compounds on the other hand may well be different. However, the tests using performic acid and sodium hydroxide do not show any marked difference in the binding *in vitro* of the hydrolysed compounds and the binding *in vivo* of the chloro compound. This suggests the possibility that, *in vivo*, the compound is largely bound after it has been hydrolysed or degraded. This hypothesis is supported by the finding that there is a different pattern of distribution of the specific radioactivities among cell fractions in the kidney from that in the liver when PAM has been injected. Furthermore, when a hydrolysate of PAM is administered it gives rise among the liver cell fractions to a pattern of specific radioactivities which resembles that of the kidney. It is clear from the experiments with plasma albumin that the products of hydrolysis of PAM are capable of being bound in some way even with soluble proteins such as plasma albumin, in a form in which they are not easily removed. Since it cannot be expected that this is a *real* " incorporation " we cannot conclude that even that part of the radioactive compound found attached to proteins in the *in vivo* experiments, which is not easily removed by performic acid, is incorporated through peptide bonds.

The present investigation gives no clear explanation of the different behaviour of PAM in its D- and L-form. However, the fact that the present investigation shows a high concentration of radioactivity to be present in the kidney, suggests that the animal is making an effort to excrete PAM or its products of degradation. It may well be that there is a difference in the rates of excretion of the D- and the L- forms. It is moreover possible that the D- compounds undergo specific changes, e.g. through the action of enzymes, such as those which act on D-amino acids or their derivatives.

SUMMARY

1. A labelled nitrogen mustard derivative of phenylalanine, *p*-di-(2-chloroethyl)amino-DL-phenyl [$\beta$-$^{14}$C] alanine (PAM), was administered to rats, some of which were bearing the Walker carcinoma.

2. The total radioactivities in the tumour, liver and blood usually had a similar level. In any experiment, the protein fraction of the kidney possessed a specific radioactivity at least three times as high as that of any other tissue examined. In the kidney the proteins of the supernatant fraction and often of the microsomes had the highest specific radioactivities. In contrast, there was little difference in the specific radioactivities of the cell fractions of the liver.

3. The proteins always had a higher specific radioactivity than the nucleic acids separated from the same tissue.

4. PAM was found to react *in vitro* with proteins of liver tissue and with plasma albumin. Radioactivity was also bound to these proteins when in the presence of hydrolysate of PAM. It follows that the mode of binding of at least part of the radioactivity derived from PAM does not require the presence of the chloro-groups and also that it is not necessarily a metabolic process.

5. Chromatography of hydrolysates of protein samples showed no radioactivity in tyrosine or phenylalanine spots so that no conversion of PAM to one of these amino acids was observed.

The author wishes to thank Professor J. A. V. Butler, F.R.S., for his advice and encouragement. He is indebted to Professor F. Bergel for helpful suggestions, to Dr. V. C. E. Burnop for making available the radioactive compound, to Dr. J. A. Stock for supplying certain nitrogen mustard derivatives and to Mrs. J. Tapley for technical assistance.

This investigation has been supported by grants to the Royal Cancer Hospital and the Chester Beatty Research Institute from the British Empire Cancer Campaign, the Jane Coffin Childs Memorial Fund for Medical Research, the Anna Fuller Fund, and the National Cancer Institute of the National Institutes of Health, United States Public Health Service.

### REFERENCES

Bergel, F., Burnop, V. C. E. and Stock, J. A.—(1955) *J. chem. Soc.*, 1223.

*Idem* and Stock, J. A.—(1953) *Ann. Rep. Brit. Emp. Cancer Campgn.*, **31**, 6.—(1954) *J. chem. Soc.*, 2409.

Borsook, H., Deasy, C. L., Haagen-Smit, A. J., Keighley, G. and Lowry, P. H.—(1952) *J. biol. Chem.*, **196**, 669.

Golombic, C., Fruton, J. S. and Bergmann, M.—(1946) *J. org. Chem.*, **11**, 518.

Jones, A. S. and Marsh, G. E.—(1954) *Biochim. biophys. Acta*, **14**, 559.

Koller, P. C. and Veronesi, U.—(1956) *Brit. J. Cancer*, **10**, 703.

Kirby, K. S.—(1956*a*) *Biochem. J.*, **62**, 31P.—(1956*b*) *Ibid.*, **64**, 405.

Peterson, E. A. and Greenberg, D. M.—(1952) *J. biol. Chem.*, **194**, 359.